# California Quicksilver

**Published:** 1851-03

**Authors:** 


### ARTICLE III.

### *California Quicksilver.*

It is not improbable that gold may be found to constitute but a small proportion of the wealth derivable from California. It has for some time past been known that quicksilver abounds in that locality to an enormous extent. About twelve months ago a capitalist embarked with the requisite machinery for working a mine, and the result has more than realized his most sanguine expectations. On his return to England in quest of additional machinery, we are informed that he found a letter from the great Rothschild—the present mercury monopolist—requesting an interview with him in London—for what purpose it is easy to guess. We believe this request was not acceeded to. The reports respecting the extent of the supply in the new mines are almost incredible. We have been informed that within a few weeks of the commencement of operations by the party alluded to, assisted by five men, a quantity of mercury was raised equal in value to £100,000 at the present market price. Even allowing a large discount for exaggeration, there is no reason to doubt that the supply is almost unlimited, and that the metal can be profitably sold at less than half its present price. The silver mines in Mexico, which have for years been unproductive, on account of the prohibitory price of mercury, may now be supplied on reasonable terms, and every branch of trade and manufacture in which mercury is used, will acquire a similar stimulus.—*Pharmaceutical Journal, Nov.* 1, 1850., *in Amer. Jour Far.*

